# Knowledge, Attitudes, and Acceptance of Genetic Engineering Among Adults in the UAE: A Cross-Sectional Study

**DOI:** 10.7759/cureus.88365

**Published:** 2025-07-20

**Authors:** Anas E Masoud, Hala A Mesmar, Musa'ab Omair, Naema M Alkaabi, Maha M Alqaydi, Manahil A Dafalla, Suni Ebby, Amal Hussein

**Affiliations:** 1 Genetics, College of Medicine, University of Sharjah, Sharjah, ARE; 2 Basic Medical Sciences, College of Medicine, University of Sharjah, Sharjah, ARE; 3 Family and Community Medicine, University of Sharjah, Sharjah, ARE

**Keywords:** attitudes, biotechnology, ethics, genetic engineering, knowledge assessment, public perception, science communication, technology acceptance, united arab emirates

## Abstract

Background: Genetic engineering has emerged as a transformative technology with significant implications for healthcare, agriculture, and environmental sustainability. Despite its potential benefits, public perception and acceptance of genetic engineering vary widely across different regions and populations. This study aims to assess the knowledge, attitudes, and acceptance of genetic engineering among adults in the United Arab Emirates (UAE).

Methods: A cross-sectional study was conducted using a self-administered questionnaire specifically designed to evaluate multiple aspects relevant to the objectives of the study. Participants were recruited through online platforms, mainly WhatsApp and LinkedIn. Eligible individuals were adults aged 20 to 50 years, fluent in Arabic or English, and residing in the UAE. Those who were nonresidents, declined consent, or lacked basic awareness of genetic engineering were excluded. The questionnaire assessed participants' knowledge of genetic engineering concepts, attitudes toward various applications, and overall acceptance of the technology. Demographic information was collected to analyze correlations with knowledge, attitudes, and acceptance levels.

Results: Out of the total 384 respondents, 300 participants met the eligibility criteria and were included in the final analysis. Results indicated that while most participants (68%) had basic knowledge of genetic engineering, a detailed understanding of specific applications was limited. Attitudes toward genetic engineering were generally positive (57%), particularly regarding medical applications, though concerns about ethical implications and safety were prevalent. Acceptance levels were moderately high (63%), with education level and prior exposure to scientific information showing significant positive correlations with acceptance. In addition, there was strong opposition to its use in nonmedical contexts, such as determining physical traits or gender. Statistically significant associations were found between knowledge and nationality (p < 0.01), occupation (p < 0.01), and income (p = 0.025), as well as between attitudes and marital status (p = 0.045) and presence of genetic disease (p < 0.01). Positive attitudes were also significantly associated with higher acceptance (p < 0.001).

Conclusion: This study highlights that the majority of adults in the UAE possess some knowledge of genetic engineering and exhibit generally positive attitudes toward its application. While the findings suggest an overall acceptance of genetic engineering among participants, there remains a mix of positive and negative perceptions influenced by various demographic and sociocultural factors. These insights can inform targeted educational initiatives and policy development to enhance public understanding and responsible implementation of genetic engineering technologies in the UAE and similar developing regions.

## Introduction

Genetic engineering has emerged as one of the most transformative scientific advancements, influencing fields such as healthcare, agriculture, and industrial biotechnology. Defined as the direct manipulation of the genetic material of an organism to achieve desired traits. Genetic engineering encompasses various techniques, including gene therapy, genome editing, and genetically modified organisms (GMOs). While these technologies hold immense potential, their acceptance among the general public is influenced by multiple factors, including knowledge levels, ethical considerations, religious beliefs, and trust in scientific institutions. Understanding public perceptions is particularly crucial in the United Arab Emirates (UAE), a rapidly advancing hub of technological innovation where cultural, ethical, and sociodemographic factors shape attitudes toward scientific advancements.

Public attitudes toward genetic engineering vary across regions and applications. Studies have found that acceptance is often highest in medical contexts, particularly when genetic engineering is used to treat or prevent diseases. For instance, research in Australia demonstrated that individuals were more receptive to genetic engineering when it had direct medical benefits rather than applications in food production [1]. Similarly, a German study highlighted that while many consumers recognized the potential benefits of genetic modification, concerns over health risks and environmental consequences played a significant role in shaping public acceptance [2]. These studies underscore the need for region-specific investigations, as perceptions of genetic engineering are influenced by cultural, educational, and religious factors.

Knowledge plays a fundamental role in shaping public attitudes toward genetic engineering. Multiple studies indicate a positive correlation between scientific literacy and acceptance of genetic technologies [3]. Research conducted among healthcare professionals in Saudi Arabia found that while knowledge about gene therapy remained limited, attitudes were largely positive, emphasizing the necessity for improved educational initiatives to enhance understanding and confidence in these technologies [4]. Likewise, a Slovakian study demonstrated that students with greater exposure to biotechnology concepts exhibited higher acceptance levels, while those with limited knowledge expressed skepticism [5]. These findings suggest that increasing genetic literacy through public education may be an effective strategy to enhance trust in genetic engineering, particularly in the UAE.

Beyond knowledge, ethical and religious considerations significantly impact public attitudes toward genetic modification. Research has shown that religious beliefs often act as a perceptual filter, influencing how individuals interpret scientific advancements [6]. In societies where religious and cultural values shape worldviews, ethical concerns regarding human intervention in natural processes often lead to resistance toward genetic engineering. For example, a study on genetically modified foods in the UK found that opposition to genetic modification was largely driven by moral concerns about "playing God" and interfering with nature [7]. Given that the UAE is deeply rooted in Islamic principles, understanding the interplay between religious beliefs and public attitudes toward genetic engineering is essential for fostering informed discussions on the ethical dimensions of these technologies.

Concerns about safety, regulatory oversight, and long-term consequences further influence public perceptions of genetic engineering. While many individuals acknowledge its potential benefits, uncertainties surrounding unintended genetic mutations, ecological imbalances, and corporate control over genetic technologies contribute to skepticism [8]. A systematic review of public perspectives regarding cell and gene therapies emphasized that while there is optimism about these advancements, regulatory clarity and transparent communication from policymakers remain crucial in building trust [9]. Addressing these concerns in the UAE through well-defined policies and public engagement initiatives will be essential for facilitating acceptance and ensuring responsible implementation.

The role of media and misinformation in shaping attitudes toward genetic engineering cannot be overlooked. A study on public knowledge of genetic testing found that while scientific literacy is gradually increasing, misconceptions remain prevalent, particularly regarding genetic risks and testing procedures [10]. In many cases, misinformation, often spread through social media, exacerbates fears about genetic technologies, leading to exaggerated concerns over their safety and ethical implications [11]. This highlights the importance of effective science communication strategies that provide accessible, transparent, and evidence-based information to the public.

Despite these challenges, research indicates that targeted educational programs and policy initiatives can significantly enhance public acceptance of genetic engineering. For instance, a study on biotechnology perceptions in Poland found that structured educational campaigns effectively improved knowledge levels and fostered more favorable attitudes toward genetic modification [12]. Similarly, research in Mali on genetically modified mosquitoes for malaria control revealed that communities were more receptive to genetic interventions when provided with transparent, locally relevant information [13]. These findings suggest that public engagement strategies that emphasize the tangible benefits and ethical considerations of genetic engineering could play a pivotal role in increasing acceptance.

These findings suggest that while public acceptance of genetic engineering is increasing, especially in medical contexts, public understanding of the science remains limited. Education and transparent communication about the benefits, risks, and regulatory safeguards are crucial to improving public perceptions and fostering trust in genetic technologies. As a center for scientific and technological innovation in the Middle East, the UAE has immense potential to leverage genetic engineering for advancements in healthcare, agriculture, and sustainability. However, public resistance or misunderstanding of these technologies could hinder their widespread adoption and implementation. Given this, the UAE serves as a valuable, unprecedented case study for examining public perceptions of genetic engineering, particularly in the context of a developing nation undergoing rapid scientific progress.

Study objectives

This study aims to contribute to the growing body of research on genetic engineering by focusing specifically on the knowledge, attitudes, and acceptance of adults in developing regions. The novelty of this research lies in its emphasis on developing countries, where public engagement with these technologies remains underexplored. The main objectives are to assess the level of public knowledge about genetic engineering in the UAE, evaluate the factors that influence public acceptance of genetic technologies, identify sociodemographic variables associated with different attitudes toward genetic engineering, and provide policy recommendations to enhance public engagement and ethical discourse around these technologies.

Additionally, our study tested two formal hypotheses. First, there is a significant association between having prior knowledge of genetic engineering and the acceptance of its application, and the second is that individuals who hold a positive impression of genetic engineering are more likely to accept its application in real-life contexts.

## Materials and methods

Study design and target population

An observational, cross-sectional study was conducted between February 2022 and January 2023 to assess knowledge, attitudes, and acceptance of genetic engineering among adult residents of the UAE. This design was chosen to provide a snapshot of public opinion at a specific point in time and identify demographic correlates without implying causality, which is a known limitation of cross-sectional surveys. Participants were recruited through convenience sampling via online platforms, primarily WhatsApp and LinkedIn. Adults aged 20 to 50 years, able to speak Arabic or English, and currently residing in the UAE were eligible for inclusion. Individuals who were not residents, unwilling to consent, or unfamiliar with the concept of genetic engineering were excluded. Due to the online recruitment strategy, we could not pre-screen or fully exclude individuals with no prior exposure to genetic modification, though this limitation was minimized through clear eligibility questions in the survey. A nonprobability sampling method was employed due to its feasibility and alignment with the data collection approach. The sample size was calculated using the formula for estimating a single population proportion:* *$n = \frac{Z^{2} \cdot p(1 - p) }{d^{2}}$, where n is the required sample size, Z is the Z-score for the desired confidence level, p is the estimated prevalence of knowledge about genetic engineering in the population, and d is the margin of error. This yielded a minimum sample size of 384, which was increased by 10% to account for nonresponses or incomplete questionnaires, resulting in a final minimum sample size of 423 participants. Ultimately, the study included 300 eligible participants who met the inclusion criteria and consented to participate. We acknowledge that the final sample was smaller than the initially calculated target, and as such, the study may be underpowered for some subgroup analyses. Confidence intervals were not calculated but are recommended for future studies. This study was reviewed and approved by the University of Sharjah Research Ethics Committee (REC-22-02-16-02-S). Participants provided informed consent electronically before proceeding with the questionnaire. The consent form clearly stated the voluntary nature of participation, the right to withdraw at any time, and the assurance of confidentiality.

Data collection tool and process

Data was collected through a self-administered questionnaire specifically designed to evaluate multiple aspects relevant to the objectives of the study. The questionnaire was adapted from existing literature and refined through expert consultation. The questionnaire was made available in both Arabic and English to accommodate the diverse population of the UAE. The translation process involved forward and backward translation by a lingual expert to ensure conceptual equivalence and cultural appropriateness. Before deployment, the questionnaire underwent pilot testing with a sample of 15 participants from the target population to assess clarity, relevance, and timing. Feedback from the pilot was used to refine the wording and structure of questions. However, formal reliability testing (e.g., Cronbach’s alpha) was not performed. The questionnaire was distributed on social media platforms such as WhatsApp and LinkedIn. It consisted of six sections: demographic information, child-related data, disease-related data, knowledge of genetic engineering, attitudes toward genetic engineering, and acceptance of its applications, as shown in the Appendix (Table 3). The demographics section gathered data on participants' age, sex, marital status, nationality, education level, occupation, monthly family income, and location within the UAE. The child-related section addressed the number of children participants had, whether any of their children had genetic diseases, and their plans for future family expansion. Disease-related data focused on participants' personal or familial history of genetic diseases, their attendance at genetic counseling sessions, and the reasons for such attendance. Knowledge was assessed through multiple-choice questions exploring participants’ understanding of genetic engineering principles, applications, and technologies. Attitudes toward genetic engineering were measured using three statements rated on a five-point Likert scale (1 = strongly disagree to 5 = strongly agree). The scores from these items were summed, resulting in a possible range from 3 to 15. Based on this range and the questionnaire design, a total score of 9 or higher was considered indicative of a positive attitude. Similarly, acceptance was assessed through eight Likert-scale questions, with summed scores ranging from 8 to 40, and a total score of 24 or above was used to reflect acceptance of genetic engineering applications. These thresholds were selected to reflect the majority agreement across items. Although cutoffs are study-specific and exploratory in nature, they provide a practical framework for analysis.

Analysis

The data collected was analyzed using IBM SPSS Statistics for Windows, Versions 26 and 28 (Released 2018 and 2021, respectively; IBM Corp., Armonk, New York, United States). Both descriptive and inferential statistical methods were employed to explore the relationships between participants' knowledge, attitudes, and acceptance of genetic engineering and their demographic characteristics. Inferential analysis was conducted to examine relationships between knowledge, attitudes, and acceptance of genetic engineering and various independent variables such as age group, sex, marital status, nationality, education level, income, occupation, and presence of genetic disease. The Chi-square test was used to assess associations between categorical variables. Additionally, the Pearson correlation coefficient was used to evaluate linear relationships between continuous and ordinal variables where appropriate. A p-value of <0.05 was considered statistically significant.

## Results

Demographics

The demographics of all 300 study participants have been summarized in Table 1. Overall, the majority of the participants were females, and the dominant age group was between 20 and 29 years of age, of which 72.80% were unmarried. More non-Emirati Arabs seemed to have participated in the study compared to UAE nationals (23.30%) and non-Arabs (6.60%). Most of the participants have either completed high school (33.90%) or have a bachelor’s degree (46.50%). In general, the participants were mostly students; however, there was a variety of monthly income responses. As expected, almost all participants did not have a genetic disease, but still, 7% of them did. Similarly, 22.30% of the respondents reported having a genetic disease in the family.

**Table 1:** Participant demographics (n = 300)

Demographic		Frequency (n)	Percent
Sex	Male	65	21.60%
	Female	235	78.40%
Age	20-29	227	75.70%
	30-50	73	24.30%
Marital status	Unmarried	218	72.80%
	Married (currently/previously)	82	27.80%
Nationality	UAE national	70	23.30%
	Other Arab	210	70.10%
	Non-Arab	20	6.60%
Education	High school	102	33.90%
	Undergraduate	139	46.50%
	Diploma	23	7.60%
	Postgraduate	36	12%
Occupation	Unemployed	26	8.60%
	Medical field employee	17	5.60%
	Nonmedical field employee	52	17.30%
	Student	205	68.40%
Income	Less than 10,000 AED	60	20.10%
	10,000 to 20,000 AED	84	27.90%
	21,000 to 30,000 AED	66	22.10%
	More than 31,000 AED	90	29.90%
Having a genetic disease	Yes	21	7%
	No	279	93%
Presence of genetic diseases in the family	Yes	67	22.30%
	No	233	77.70%

Knowledge of genetic engineering

**Figure 1:**
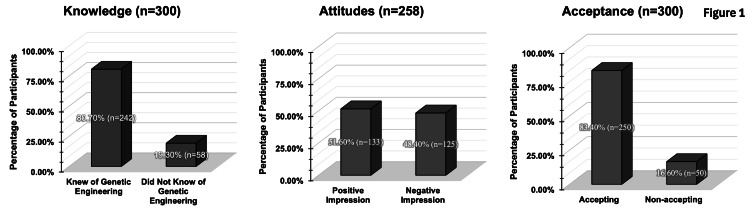
Knowledge is defined by having heard of the concept of genetic engineering. Attitudes toward genetic engineering were grouped into positive and negative, excluding those who answered with "neutral" to most questions when asked. Acceptance of the concept of genetic engineering was according to the questionnaire scoring system

Figure 1 shows knowledge of, attitudes toward, and acceptance of genetic engineering. A total of 80.70% of the participants knew of its existence. Regarding the knowledge part of the questionnaire, we asked the participants if they had heard of the term "genetic engineering," what sources had helped them to get to know genetic engineering, the number of sources that introduced them to this knowledge, and finally, we asked them to choose the right definition for this term. Regardless of the sources used and the number of sources that introduced the participants to this technology, knowledge in our questionnaire was determined by the number of people who chose "yes" when asked if they had ever heard of genetic engineering. This decision was made to allow for consistent statistical analysis. However, we acknowledge that this method may not fully capture participants’ actual understanding, as some individuals who responded “no” were still able to select the correct definition. Ideally, a composite knowledge score based on the number of correct responses would have provided a more accurate reflection of participant knowledge.

Attitudes toward genetic engineering

As shown in Figure 1, nearly half of the participants (51.60%) had a positive impression of genetic engineering. When questioning the attitudes of the adults in the UAE toward genetic engineering, we first asked them a general question, how they felt about this term, in which almost half of the participants agreed that it felt understandable enough. Then, we implemented a scoring system for three questions that asked if (1) genetic engineering can make lives easier, (2) it provides opportunities for discovery, and (3) genetic engineering could be beneficial. Participants who agreed or strongly agreed with all three statements were classified as having a positive attitude toward genetic engineering. Those who disagreed or strongly disagreed with most of these items were classified as having a negative attitude. Neutral responses were excluded from this categorization. Using this approach, 51.60% of participants were categorized as holding positive attitudes.

Acceptance of genetic engineering

Figure 1 also shows that 83.40% of the population seems to be accepting of the concept of implementing genetic engineering into real-life use. A similar scoring system was used for the acceptance part. We determined the level of acceptance in the population by directly giving them five-point Likert scale questions, and then, we calculated acceptance by summing responses indicating agreement or strong agreement and applied a cutoff point, whereby participants scoring above the threshold were classified as accepting the technology overall. While acceptance of genetic engineering was clear, a good majority refused its implementation to determine the characteristics of fetuses. For example, 70% of the population refused the use of genetic engineering for determining gender, 85% refused to use it to change the physical features of fetuses, and more than 80% of them admitted being afraid that genetic engineering would be used in nonmedical situations where it is not needed or might pose a threat. On the other hand, most acceptance was toward using genetic engineering in situations like curing and preventing diseases.

Correlations of attitudes and knowledge with independent variables

Table 2 summarizes the significant associations found when we performed the Chi-square test with several independent variables to find the factors affecting the knowledge, attitudes, and acceptance of genetic engineering in the population of the UAE. The table includes only the significant associations (p < 0.05), in which several variables seemed to have influenced, especially the attitudes and knowledge of the population regarding genetic engineering.

**Table 2:** Correlates with attitudes and knowledge using Chi-square, where the statistical significance threshold is p < 0.05

Independent variable	Attitudes	Knowledge
Nationality		p < 0.01
Having a genetic disease	p < 0.01	
Income		p = 0.025
Marital status	p = 0.045	
Occupation		p < 0.01
Acceptance	p < 0.001	

The study shows that there is a significant relationship between knowledge and nationality. Non-Emirati Arabs seem to have known about genetic engineering more than other nationalities. There is also a correlation between monthly income and knowledge about genetic engineering, in which 51.7% of the participants who knew of it were also those with high monthly income, as compared to the ones who denied knowing it, who were of low income. Moreover, occupation was linked to knowledge. According to the study, students seemed to represent most of the population that knew of genetic engineering (84.5%), revealing that students are almost two times more likely to know about genetic engineering in comparison to employees of other occupations.

As for attitudes, having a genetic disease and attitudes toward genetic engineering were correlated, however, paradoxically. Of those who had a genetic disease, most of them (77.8%) showed a negative impression toward the future use of genetic engineering. In comparison, for those who do not have a genetic disease, nearly half showed a positive impression of genetic engineering implementation. The study also showed that unmarried individuals are more positive toward the use of genetic engineering compared to the currently or previously married participants, in which it was shown that unmarried respondents were 1.77 times more likely to show positive impressions of genetic engineering than married participants. Moreover, there was an association between attitudes toward and acceptance of genetic engineering. The participants who had a positive impression of genetic engineering were more accepting of using genetic engineering than those who had a negative impression of it.

## Discussion

To our knowledge, this is the first study that has been done to provide insight into the level of acceptance, attitudes, and knowledge toward genetic engineering among the adult age group in the UAE. Moreover, our study has shown that the acceptance of the concept of genetic engineering is highly dependent on the intended application. Consistent with previous research, such as that of Akatsuka et al. [14], which showed that Japanese participants were more willing to accept germline genome editing for disease treatment (around 50-63%) than for basic research (around 39-43%). Our study found a similar conditional acceptance. In the UAE, a country where Islamic values, conservative ethical norms, and evolving regulatory frameworks play a central role, public support was primarily focused on medical applications such as disease treatment and prevention, while the use of genetic engineering for cosmetic or nonmedical purposes was widely rejected.

Based on 300 respondents, our findings indicate that most of the participants disagreed or strongly disagreed with the idea of altering the characteristics of embryos using genetic engineering, irrespective of the cause. This is considered a significant result that goes in line with Weisberg et al. [15], which demonstrated that more than half of the study population who answered with “acceptable depending on the purpose” were for the questions regarding germline genome editing for disease research.

In addition, our study tested two main hypotheses. The first examined whether there was a correlation between the application of genetic engineering and having prior knowledge of it. We found no meaningful association between the two, suggesting that simply being aware of genetic engineering did not significantly influence participants’ acceptance of its use. The second hypothesis explored whether individuals’ impressions of genetic engineering were linked to their acceptance of its application. In this case, we did find a clear relationship, as participants who had a more positive impression of genetic engineering were more likely to support its use.

Furthermore, our research indicated that factors such as gender, age, and attending genetic counseling sessions were found to be insignificant as to differences in acceptance. On the other hand, the results of Akatsuka et al. and Delhove et al. [14,16] showed that gender and age played different important roles in the acceptance and support of the technology. Women were more resistant to the acceptance of genetic engineering, and the younger population was more supportive of the technology as opposed to the older age group. However, our analysis is consistent with other studies in which parents of children who have genetic diseases, compared to parents who do not, are more inclined to accept the use of the technology to genetically modify embryos [16]. However, as the number of participants with children with genetic diseases (n = 6) is low, this statement may be biased.

We hypothesized that the monthly income, occupation, and nationality of the participants would affect the level of knowledge of the technology. With this information presented, results showed that UAE nationals, those on the higher side of income, and those employed in the medical field have more knowledge about genetic engineering compared to Arabs and non-Arabs, those in the lower income groups, and employees outside the medical field. A correlation between nationality and occupation to high income can be assumed, which may explain the findings, but further investigation may be required to assess disparities. In addition, education played a significant role in shaping public acceptance, with the majority of tertiary-educated individuals endorsing medical applications of the technology.

The knowledge part of the questionnaire contained a couple of questions, including having heard of the concept of genetic engineering, choosing the correct definition of the term "genetic engineering," which more than half of the participants have chosen correctly, and the sources where they were introduced to genetic engineering. To determine the level of knowledge, we decided that having heard of the concept of genetic engineering is enough, since some respondents managed to choose the correct answer regarding the definition, regardless of having heard of genetic engineering or their different sources of knowledge.

Regarding attitudes, we implemented a scoring system according to the responses in the "attitudes" part of the questionnaire. When asked about their impression of the term "genetic engineering" almost half of the respondents chose that it feels understandable, and a minority of them confessed they did not know how to feel about it or that it feels confusing to them. We then asked them several questions using the five-point Likert scale and gave the responses in the scale numbers, which we added later on to make a scoring system for attitudes towards genetic engineering. Most of the population has agreed/strongly agreed that genetic engineering can be used to make human life easier with its various applications. An overwhelming percentage of the participants have yet again chosen "agree" to "strongly agree" when asked if genetic engineering can provide opportunities for discoveries. Most of the participants also agreed that genetic engineering is beneficial.

In the bivariate analysis of attitudes toward genetic engineering and the two variables of marital status and having a genetic disease, we found both relationships to be highly significant. More than half of unmarried participants showed positive attitudes toward genetic engineering. These findings suggest a relatively moderate level of optimism among younger or unmarried UAE adults. Interestingly, this aspect of marital status influencing attitudes toward genetic engineering is underexplored in previous research. Most existing studies focus on factors such as age, gender, education, and income but do not specifically examine the role of marital status. Therefore, our study adds a novel perspective that warrants further investigation to understand how marital status and related experiences shape public perceptions of genetic technologies, especially in the UAE cultural context. This also reflects the UAE’s growing focus on biotechnology and innovation, which may contribute to increased public familiarity and openness to emerging technologies. The more reserved attitudes among married individuals could relate to greater exposure to practical concerns, such as reproductive risks or ethical issues discussed during genetic counseling. The same can be said about participants with genetic diseases themselves, as more than two-thirds of this group expressed negative attitudes toward the technology. This may also be attributed to prior personal or familial experiences with the limitations or challenges of genetic conditions, potentially shaping their skepticism toward new interventions. Further investigation is required to fully understand these associations.

Study limitations

This study, while offering important insights into public perceptions of genetic engineering in the UAE, has several limitations that should be considered when interpreting the results. First, the use of nonrandom convenience sampling through online platforms such as WhatsApp and LinkedIn likely introduced selection bias. This approach disproportionately captured younger, digitally active individuals, particularly females and university students, and may not reflect the broader UAE population. While this method allowed for timely data collection and ensured accessibility across different Emirates, future studies should consider employing stratified or random sampling to enhance representativeness. Second, self-reported data, whether collected online or in person, carry the risk of social desirability bias, where participants may respond in ways that align with perceived societal expectations. This may especially affect attitudes and acceptance levels. Although anonymity was preserved to reduce this effect, it cannot be entirely ruled out. Third, although the questionnaire was reviewed for content validity by an expert and made available in both Arabic and English, it was not formally tested for psychometric properties such as internal consistency (e.g., Cronbach’s alpha). These factors may limit the robustness of the tool used to assess knowledge, attitudes, and acceptance. Nonetheless, the scoring system was based on established formats in previous literature and tailored to the UAE context. Fourth, while we calculated a required sample size of 384 participants (with an adjusted target of 423 to account for nonresponses), only 300 valid responses met eligibility criteria and were included in the final analysis. This shortfall may reduce the statistical power of some findings and explains why confidence intervals were not reported. However, the achieved sample size is consistent with or exceeds many similar exploratory studies in the region and still allows for meaningful initial conclusions. Fifth, the study’s statistical analysis was limited to Chi-square and Pearson correlation tests without the inclusion of confidence intervals or effect size measures. While these tests were appropriate for the nature of our variables and study objectives, the absence of more advanced analytical methods and test statistics may restrict the depth of interpretation. As such, the strength and direction of associations should be interpreted with caution, particularly given the study's sample size and cross-sectional design. Sixth, a cross-sectional design inherently limits our ability to infer causal relationships. While correlations between variables such as marital status and attitudes were identified, no temporal sequence or causality can be assumed. This is a recognized limitation of cross-sectional surveys and highlights the need for longitudinal or qualitative follow-up studies. Finally, although the cultural and religious context of the UAE is recognized as a potential influencer of attitudes toward biotechnology, these variables were not explicitly measured in our questionnaire. Including such measures in future studies could deepen understanding of how these social factors shape public opinion on emerging technologies. Despite these limitations, this study provides novel and regionally relevant data from a previously understudied population. The findings contribute to a foundational understanding of how adults in the UAE perceive genetic engineering, offering a baseline for future research, public health initiatives, and policy development.

## Conclusions

This study highlights that the majority of adults in the UAE possess some knowledge of genetic engineering and exhibit generally positive attitudes toward its application. While the findings suggest an overall acceptance of genetic engineering among participants, there remains a mix of positive and negative attitudes, reflecting a complex public perception. Knowledge of genetic engineering was more prevalent among individuals with higher socioeconomic status, particularly those with access to education. Despite this awareness and the high levels of acceptance, the study encountered limitations that hindered a definitive conclusion on the necessity for raising further public awareness.

Given that this research is the first of its kind in the UAE, the results underscore the importance of additional investigations. Future studies should aim to include a larger and more diverse sample, especially older adults, to gain a comprehensive understanding of public attitudes and knowledge. Moreover, incorporating physical interviews in future research would be essential in minimizing bias and improving the accuracy of public opinion assessments on genetic engineering. Such efforts will contribute to a deeper understanding of societal perspectives on these rapidly advancing biotechnological applications.

## Appendices

**Table 3:** English version of the self-administered questionnaire to assess the knowledge, attitudes, and acceptance of genetic engineering among adults in the UAE This questionnaire was developed by the authors of this study after reviewing the literature about the topic. Reviewed for scientific accuracy by Sunni Ebby

Category	Question	Answer options
Demographics	D1. What is your age group?	20-29 years
		30-39 years
		40-50 years
	D2. Gender	Male
		Female
	D3. Marital status	Not married
		Married
		Was married
	D4. Nationality	Local
		Arab resident
		Non-Arab resident
	D5. What is the name of the city where you currently live?	Abu Dhabi
		Dubai
		Sharjah
		Ajman
		Ras al kahima
		Fujairah
		Um al-Quwain
	D6. What is the highest level of education you have reached?	Less than year 12
		Highschool
		Undergraduate
		Diploma
		Post-graduate
	D7. Current occupation	Specify: _____________
	D8. Monthly family income	Less than 10,000 AED
		11,000 to 20,000 AED
		21,000 to 30,000 AED
		More than 30,000 AED
Child related data	C1. Do you have any children?	Yes (answer C2)
		No (skip to C3)
	C2. How many children do you currently have?	1-3
		4-6
		More than 6
	C3. Do you plan on having (more) kids in the future?	Yes
		No (skip C4)
	C4. Does anyone of your children have any genetic disease?	Yes (specify: _____________)
		No
Disease related data	D1.Does anyone from your family (parents/siblings) have a genetic disease?	Yes (specify: _____________)
		No
	D2. Do you have any genetic diseases?	Yes (specify: _____________)
		No
	D3. Have you ever attended a genetic counseling session?	Yes (answer D4)
		No (skip D4)
	D4. Why did you attend a genetic counseling session? (Select all that applies)	Because I wanted to know if I am at possible risk of a genetic disease
		Because I already have a genetic disease
		Because I was planning to have a child
		Because I already have a child with a genetic disease
		Because I had a miscarriage
		Other: specify: _____________
Knowledge of genetic engineering	K1. Have you ever heard about the term ‘genetic engineering’?	Yes
		No
	K2. If ‘yes’, where did you get your information?	Social media
		Internet
		Medical professional
		School education
		Other (specify: _____________)
	K3. What is genetic engineering?	It searches the genetic material’s structure and function
		Use of genetic material in different applications
		It examines heredity and variation
		Using biotechnology to change the genes of an organism
	K4. What is your impression of the term “genetic engineering”?	Feels confusing
		Feels understandable
		I Don’t know
	K5. Genetic engineering studies with animals are beneficial to people	Strongly agree
		Agree
		Neutral
		Disagree
		Strongly disagree
	K6. Organisms with altered genes contain risks for nature	Strongly agree
		Agree
		Neutral
		Disagree
		Strongly disagree
	K7.Genetic engineering can provide opportunities for new discoveries	Strongly agree
		Agree
		Neutral
		Disagree
		Strongly disagree
	K8. Genetic engineering makes human life easier	Strongly agree
		Agree
		Neutral
		Disagree
		Strongly disagree
Acceptance of genetic engineering	A1. How much do you agree with the use of genetic editing of cells in children or adults to cure a life-threatening disease? This means the disease could still be passed on to their children.	Strongly agree
		Agree
		Neutral
		Disagree
		Strongly disagree
	A2. How much do you agree with the use of genetic editing of cells in embryos to prevent a life-threatening disease?	Strongly agree
		Agree
		Neutral
		Disagree
		Strongly disagree
	A3.How much do you agree with the use of genetic editing of cells in embryos to alter any non-disease characteristic - such as memory, eye color or height? This would mean that all subsequent generations would have the same genetic characteristics.	Strongly agree
		Agree
		Neutral
		Disagree
		Strongly disagree
	A4. How much would you agree with the use of gene editing to change a baby’s genetic characteristics if it required testing on human embryos?	Strongly agree
		Agree
		Neutral
		Disagree
		Strongly disagree
	A5. If you could safely genetically edit your embryo would you use this technology to determine physical appearance (eye color; hair color; skin color)?	Yes
		No
	A6. If you could safely genetically edit your embryo would you use this technology to determine the gender of your baby?	Yes
		No
	A7. If you could safely genetically edit your embryo would you use this technology to determine intelligence?	Yes
		No
	A8. How much do you agree with this statement “I fear that gene editing might be used in non-medical circumstances”	Strongly agree
		Agree
		Neutral
		Disagree
		Strongly disagree
